# Heavy strength training improves running and cycling performance following prolonged submaximal work in well‐trained female athletes

**DOI:** 10.14814/phy2.13149

**Published:** 2017-03-14

**Authors:** Olav Vikmoen, Bent R. Rønnestad, Stian Ellefsen, Truls Raastad

**Affiliations:** ^1^ Section for Sport SciencesLillehammer University CollegeLillehammerNorway; ^2^ Deparment of Physical PerformanceNorwegian School of Sport SciencesOsloNorway

**Keywords:** Concurrent training, cycling economy, prolonged cycling, prolonged running, running economy

## Abstract

The purpose of this study was to investigate the effects of adding heavy strength training to female duathletes' normal endurance training on both cycling and running performance. Nineteen well‐trained female duathletes (*V*O_2max_ cycling: 54 ± 3 ml∙kg^−1^∙min^−1^, VO_2max_ running: 53 ± 3 ml∙kg^−1^∙min^−1^) were randomly assigned to either normal endurance training (*E*,* n* = 8) or normal endurance training combined with strength training (*E+S*,* n* = 11). The strength training consisted of four lower body exercises [3 × 4‐10 repetition maximum (RM)] twice a week for 11 weeks. Running and cycling performance were assessed using 5‐min all‐out tests, performed immediately after prolonged periods of submaximal work (3 h cycling or 1.5 h running). *E+S* increased 1RM in half squat (45 ± 22%) and lean mass in the legs (3.1 ± 4.0%) more than *E*. Performance during the 5‐min all‐out test increased in both cycling (7.0 ± 4.5%) and running (4.7 ± 6.0%) in *E+S,* whereas no changes occurred in *E*. The changes in running performance were different between groups. *E+S* reduced oxygen consumption and heart rate during the final 2 h of prolonged cycling, whereas no changes occurred in *E*. No changes occurred during the prolonged running in any group. Adding strength training to normal endurance training in well‐trained female duathletes improved both running and cycling performance when tested immediately after prolonged submaximal work.

## Introduction

During the last decade, increased attention has been given to the effects of adding strength training to endurance athletes' normal training on running and cycling performance (e.g., Paavolainen et al. 1999; Aagaard et al. 2011; Ronnestad et al. 2011; Sedano et al. 2013). Improvements in performance have been reported in both running (Paavolainen et al. 1999; Storen et al. 2008; Sedano et al. 2013; Damasceno et al. 2015) and cycling (Koninckx et al. 2010; Ronnestad et al. 2010a; Aagaard et al. 2011; Ronnestad et al. 2015). However, the literature is far from conclusive, and numerous studies do not report such improvements in neither running (Ferrauti et al. 2010; Roschel et al. 2015) nor cycling (Bishop et al. 1999; Bastiaans et al. 2001; Levin et al. 2009). Some methodological differences may explain these equivocal findings. To positively affect cycling performance, it seems that the strength training regime needs to involve heavy loads**,** typically between 10 and 4 repetition maximum (RM) (Koninckx et al. 2010; Ronnestad et al. 2010a; Aagaard et al. 2011; Ronnestad et al. 2015). To improve running performance on the other hand, both explosive, plyometric and heavy strength training seems effective (Paavolainen et al. 1999; Sedano et al. 2013; Damasceno et al. 2015). To the best of our knowledge, only one study has investigated the effect of strength training on performance in both cycling and running in the same athletes. This study reported increased time to exhaustion at *V*O_2max_ in both cycling and running (Hickson et al. 1988). However, the study did not include an endurance training only group, and therefore the results should be interpreted with caution.

The observation that somewhat different strength training regimes affect performance in cycling and running indicates that some of the performance‐enhancing mechanisms may differ between these sports. Suggested mechanisms by which strength training can improve cycling and running performance include changes in rate of force development, changes in tendon stiffness, changes in movement mechanics, and changes in muscular characteristics such as increased muscle strength, muscle mass, and improved anaerobic capacity (Saunders et al. 2006; Ronnestad and Mujika 2014). Some of these factors may be important for performance in both running and cycling, whereas other mechanisms may affect performance differently in these sports. For example, in running, the stretch‐shortening cycle in each stride enables the possibility to store and recoil elastic energy, whereas in cycling, the possibilities to take advantage of stored elastic energy is negligible. Consequently, a factor such as muscle‐tendon stiffness may play a role for running performance, but likely not for cycling performance. On the other hand, a factor like improved anaerobic capacity should affect performance to the same degree in both running and cycling.

Road races in cycling often consist of a long initial period of cycling at a moderate intensity, followed by an all‐out performance at the end. Even though running competitions are ran at a more even pace, they are also often decided with an all‐out effort in the end. During such efforts, a quite large proportion of the energy demand will come from anaerobic sources (Gastin 2001). Therefore, performance during a relatively short test will in addition to VO_2max_ and other aerobic parameters also be largely influenced by anaerobic capacity. Muscle mass is an important determinant of anaerobic capacity (Bangsbo et al. 1993). We have previously reported increased CSA of *m. quadriceps femoris* after 11 weeks of heavy strength training in female endurance athletes together with increased mean and peak power during the Wingate test (Vikmoen et al. 2016a). This indicates improved anaerobic capacity in the same athletes included in this study. Therefore, performance in a quite short performance test should be positively affected by this strength training regime. In addition to increased muscle CSA, changes in protein levels and expression of genes coding for proteins that are involved in the anaerobic metabolism might contribute to increased anaerobic performance.

Performance in an all‐out effort at the end of long competitions should also be affected by the fatigue developed during the competition. In Ronnestad et al. (2011), such performance was simulated by 3 h of submaximal cycling followed by a 5‐min all‐out test. Power output during the 5‐min all‐out test was improved following 12 weeks of heavy strength training in well‐trained male cyclists. This was related to improved cycling economy and reduced physiological strain during the final hour of the submaximal trial, leaving the strength‐trained athletes less fatigued before the 5‐min all‐out test (Ronnestad et al. 2011). However, no previous study has assessed effects of heavy strength training on all‐out performance following a prolonged submaximal work or physiological responses during prolonged submaximal running.

The primary purpose of this study was to investigate the effects of 11 weeks of heavy strength training on 5‐min all‐out performance after separate trials of prolonged submaximal work in both running and cycling and on physiological responses during the prolonged work. We especially wanted to identify performance‐enhancing mechanisms after strength training which acts similarly and differently on cycling and running performance.

We hypothesized that the addition of heavy strength training would result in improved 5‐min all‐out performance in both cycling and running. Furthermore, we hypothesized that changes in 5‐min all‐out performance would be related to improved work economy during the prolonged trials and to changes related to anaerobic capacity such as increased muscle mass and changes in expression of genes that are involved in anaerobic processes. We also anticipated that some of the underlying mechanisms for improved work economy would differ between running and cycling.

## Methods

### Ethical approval

The study was approved by the Local Ethics Committee at Lillehammer University College. Written informed consent was obtained from all athletes prior to inclusion, and the study was carried out in accordance with the Declaration of Helsinki.

### Participants

Twenty‐eight female duathletes who fulfilled at least two of Jeukendrup et al. (2000) training and race status descriptions of a well‐trained athlete were recruited to this study. None of the athletes had performed systematic strength training for the last 12 months leading up to the study. The athletes were matched on *V*O_2max_ and randomly assigned to either adding heavy strength training to the ongoing endurance training (*E* *+ S*,* n* = 14) or endurance training only (*E*,* n* = 14). During the study, three athletes in *E* *+* *S* left the project for reasons unrelated to the project protocol: one because of an injury, one because of a prolonged period of illness during the last part of the intervention and one because of other medical reasons. In *E,* six athletes left the study for reasons unrelated to the project protocol (injuries from bicycle crash, pregnancy, and lack of time). Therefore, the final numbers of athletes in *E* *+* *S* and *E* were 11 and 8, respectively.

### Experimental overview

This study is part of a larger study investigating the effects of heavy strength training on various aspects of cycling and running performance. The effect on time‐trial performance and traditional performance determinants in cycling and running has been previously reported (Vikmoen et al. 2016a,b). Whenever data from these studies are utilized for correlation purposes or otherwise, it will be clearly specified. The strength training program for the *E+S* group consisted of two strength training sessions per week and lasted for 11 weeks (during the competition period from April to July). The testing before and after the intervention period was organized in five test days. During pretests, test day 1 consisted of biopsy sampling from *m. vastus lateralis* for determination of muscle fiber type composition and mRNA expression of genes related to fat and anaerobic metabolism. Test day 2 consisted of a *V*O_2max_ test in cycling followed by 1RM test in half squat. Test day 3 consisted of a *V*O_2max_ test in running. Test day 4 consisted of a prolonged submaximal running trial followed by a 5‐min all‐out test. Test day 5 consisted of a prolonged submaximal cycle trial followed by a 5‐min all‐out test. There were at least 7 days between day 1 and 2 and 3–7 days between the remaining test days. After the intervention period, the only difference in test order was that muscle biopsies were taken on the last test day.

### Training

Endurance training duration and intensity were calculated based on heart rate (HR) recordings. Endurance training was divided into three HR zones: (1) 60%–82%, (2) 83%–87%, and (3) 88%–100% of maximal HR. For detailed information on endurance training characteristics, see Vikmoen et al. (2016a). Briefly, there were no significant differences between groups in their average weekly endurance training duration or distribution between intensity zones.

The heavy strength training for the *E* *+* *S* groups targeted leg muscles and were performed twice per week during the 11‐week intervention period. Adherence to the strength training was high, with *E* *+* *S* athletes completing 21.4 ± 1.0 (range 19–22) of the planned 22 strength training sessions. The strength training program was performed as reported in Vikmoen et al. (2016a). Briefly, each strength training session consisted of four leg exercises: half squat in a smith machine, leg press with one leg at a time, standing one‐legged hip flexion, and ankle plantar flexion. Three sets were performed *per* exercise. An investigator supervised the athletes at all workouts during the first 2 weeks and at least one workout per week thereafter. During weeks 1–3, athletes trained with 10RM sets at the first session and 6RM sets at the second session. These alternating loads were adjusted to 8RM and 5RM during weeks 4–6, and was further adjusted to 6RM and 4RM during weeks 7–11. The athletes were encouraged to increase their RM loads continually throughout the intervention period and they were allowed assistance on the last repetition.

### Physical performance tests

The athletes were instructed to refrain from intense exercise the day preceding testing and to prepare for the tests as they would have done for a competition. This included consuming the same type of meal at the same time as they would do if the test was a regular competition. Furthermore, the participants were instructed to replicate the preparation before every test. All cycling tests were performed on a electromagnetically braked cycle ergometer (Lode Excalibur Sport, Lode B. V., Groningen, The Netherlands), which was adjusted according to each athlete preference for seat height, horizontal distance between tip of seat and bottom bracket, and handlebar position. During all cycling tests the ergometer was in a cadence‐independent mode (constant watt‐production); so, the power output was not affected by the cyclists' chosen cadence. The running tests were performed on a motor‐driven treadmill (Woodway Desmo Evo, Waukesha, WI). The inclination of the treadmill was set to 5.3% at all tests. All testing were performed under similar environmental conditions (18–20°C).

#### *V*O_2max_ in cycling

The cycling *V*O_2max_ test protocol utilized in this study and its results has been described elsewhere (Vikmoen et al. 2016a). Briefly, the test was initiated with 1‐min cycling at a power output of 100 W that was subsequently increased by 25 W every minute until exhaustion. *V*O_2_ was measured (30‐sec sampling time) using a computerized metabolic system with mixing chamber (Oxycon Pro, Erich Jaeger, Hoechberg, Germany). The gas analyzers were calibrated with certified calibration gases of known concentrations before every test. The flow turbine (Triple V, Erich Jaeger, Hoechberg, Germany) was calibrated before every test with a 3 l, 5530 series, calibration syringe (Hans Rudolph, Kansas City, USA). *V*O_2max_ was calculated as the average of the two highest 30 sec *V*O_2_ measurements. Peak cycling performance during the test (W_max_) was calculated as the mean power output during the last 2 min of the incremental test. After the test, blood [la^−^] and HR_peak_ was noted. [La^−^] were analyzed in whole blood with a Lactate Pro LT‐1710 analyzer (Arcray Inc., Kyoto, Japan). RPE was recorded using the Borg scale (Borg, 1982). HR was measured using a Polar S610i heart rate monitor (Polar, Kempele, Finland).

#### Prolonged submaximal cycling followed by a 5‐min all‐out cycling test

The prolonged cycling lasted for 180 min on a power output corresponding to 44% of W_max_ (111 ± 9 W and 116 ± 8 W in *E* *+* *S* and *E,* respectively). The same absolute power output was utilized post intervention. *V*O_2_ and HR were determined during 3‐min periods every 30th min throughout the prolonged cycling and RPE and [la^−^] were measured every 30th min. Average values for each hour were calculated and used for statistical analyses. Athletes were allowed to occasionally stand in the pedals during the prolonged cycling, but not during the 3‐min periods of measurements and not during the final 5‐min all‐out test. Athletes were allowed to consume water and a sport drink containing 60 g/L carbohydrates, ad libitum, in order to maintain fluid balance and mimic race conditions. The amount of sport drink consumed were similar between groups and from pre to post (across groups, values were 1.24 ± 0.57 L and 1.26 ± 0.59 L, respectively). After conclusion of the prolonged cycling, athletes were allowed a 3‐min rest before a 5‐min all‐out test for determination of cycling performance. During the first minute of the test, the power output was set by the investigators. This individual selected power output was based on pilot work and corresponded to 85% of W_max_. Thereafter, the control unit for the power output was put next to the ergometer and the athletes were allowed to adjust the power output themselves with the instruction to cycle at the highest average power output as possible. The participant received feedback regarding power output and elapsed time, but not HR or cadence. Performance was measured as the mean power output during the 5‐min all‐out test. At the posttest, one athlete in *E +* *S* had to withdraw during the prolonged test due to pain in the hip. Therefore, the final numbers included in the statistical analysis of these tests are 10 in *E +* *S* and 8 in *E*.

#### *V*O_2max_ in running

The *V*O_2max_ test protocol utilized in this study and its results have been described elsewhere (Vikmoen et al. 2016b). Briefly, the test was initiated with 1‐min running at 8 km·h^−1^ that was subsequently increased by 1 km·h^−1^ every minute until exhaustion. *V*O_2max_ was calculated as the average of the two highest 30 sec *V*O_2_ measurements. Peak running performance during the test (*V*_max_) was calculated as the mean running velocity during the last 2 min of the incremental test.

#### Prolonged submaximal running followed by a 5‐min all‐out running test

The prolonged running lasted for 90 min at a speed corresponding to 60% of *V*_max_ (7.7 ± 0.4 km·h^−1^ and 7.9 ± 0.3 km·h^−1^ in *E + S* and *E,* respectively). Each participant ran at the same absolute speed at both pretrial and posttrial. *V*O_2_ and HR were measured during 3‐min periods every 15th min throughout the prolonged running and RPE and [la^−^] were measured every 15th min. Average values for each 30‐min period were calculated and used for statistical analyses. The athletes were allowed to consume water and a sport drink containing 60 g·L^−1^ carbohydrates, ad libitum, in order to maintain fluid balance. The amount of sport drink consumed was similar between groups and from pre to post (across groups values were 0.76 ± 0.27 L and 0.72 ± 0.24 L, respectively). After conclusion of the prolonged running, the athletes were allowed a 3‐min rest before a 5‐min all‐out test was performed for determination of running performance. During the first minute of the test, the speed was set by the investigators. This individual selected speed was based on pilot work and corresponded to 85% of *V*_max_. Thereafter, the athletes were allowed to adjust the speed themselves with the instruction to run as fast as possible. The athletes received feedback on speed and elapsed time, but not HR or distance. Performance was measured as the distance covered during the 5‐min all‐out test.

#### 1RM tests

Approximately 20 min after termination of the cycling *V*O_2max_ test, maximal strength in the legs was tested as 1RM in half squat. The 1RM protocol used has been described elsewhere (Vikmoen et al. 2016a). Briefly, the 1RM test started with a specific warm‐up, consisting of three sets with gradually increasing load (40, 75, and 85% of expected 1RM) and decreasing number of repetitions (10→6→3). The first attempt was performed with a load approximately 5% below the expected 1RM. If a lift was successful, the load was increased by approximately 5%. The test was terminated when the athletes failed to lift the load in 2–3 attempts and the highest successful load lifted was noted as 1RM. Athletes were given a 3‐min rest between lifts.

### Lean mass in the legs

Lean mass in the legs (Leg_LM_) was determined by dual‐energy X‐ray absorptiometry using a Lunar Prodigy densiometer (Prodigy Advance PA+302047, Lunar, San Francisco, CA, USA). The athletes were instructed to refrain from training for the 24 h leading up to the measurement. They were also instructed to not ingest any food or liquid for the 3 h preceding the measurement. The same trained technician performed all DXA scans on each participant. Care was taken to position the body at the same location at each measurement.

### Muscle biopsy sampling

Muscle biopsies were sampled from *m. vastus lateralis* using the Bergström procedure and treated as previously described (Vikmoen et al. 2016a). An appropriately sized muscle sample was excised and selected for quantitative real‐time PCR (qRT‐PCR) analyses (average wet weight ± SD: 38 ± 7 mg), and a similarly sized sample was selected for immunohistochemical analyses (average wet weight ± SD: 34 ± 13 mg). Pre‐ and post‐biopsies were sampled at the same time of day for each particular athlete. Athletes were instructed to refrain from physical activity during the last 24 h before biopsy sampling and not to ingest any food the 3 h preceding the biopsy. Biopsies for qRT‐PCR analyses were immersed immediately in RNA*later* ^®^ and treated according to manufacturers' protocol before storage at −80°C (Ambion, Foster City, CA). Biopsies for immunohistochemical analyses were formaldehyde fixated (Chemi‐teknik AS, Oslo, Norway).

### Muscle biopsy analyses

#### Immunohistochemistry

Protocols for immunohistochemical analyses of muscle fiber type composition and the results have been presented elsewhere (Vikmoen et al. 2016a). Briefly, formalin‐fixed muscle biopsies were paraffin‐embedded and sectioned, whereupon transverse, serial sections were labeled for MyHCI (*A4.840, H. Blau, Stanford, USA; Developmental Studies Hybridoma Bank)*, MyHCIIA (*EPR5280, Nordic Biosite*), and MyHCIIX (*6H1, C Lucas, Sydney, Australia; Developmental Studies Hybridoma Bank*). Determination of muscle fiber composition was performed using Photoshop CS6 Extended (Adobe, San Jose, CA). The investigator performing the image analyses were blinded as to which group the athlete belonged. Muscle fibers that were positive for both MyHCIIA and MyHCIIX are referred to as muscle fiber type IIAX‐IIX (Vikmoen et al. 2016a). Because of technical problems with some analyses, the number of individuals in the immunohistochemistry data is eight in *E + S* and eight in *E*.

#### Gene expression

Gene expression was assessed for genes involved in fatty acid oxidation and anaerobic energy metabolism. Primer design, RNA extraction, quantitative PCR (qPCR), and evaluation of the stability of reference genes was performed as previously described (Ellefsen et al. 2014). *β*_2_‐microglobulin and ribosomal protein L32 were found to be the two most stable references genes and were utilized for calculation of normalization factors using GeNorm, which were in turn utilized for calculation of target gene expression. All genes with associated primers are presented in Table 1.

**Table 1:** Details of primers used for RT‐qPCR

Gene	Forward primer	Reverse Primer
LDHA^a^	ATTCAGCCCGATTCCGTTAC	TTCCACTCCATACAGGCACAC
LDHB^a^	CATGGATGGATTTTGGGGGAAC	AACACCTGCCACATTCACAC
MCT1^a^	TTGGAGTCATTGGAGGTCTTGG	CCAATGGTCGCCTCTTGTAG
MCT4^a^	AGGCAAACTCCTGGATGCG	AAAATCAGGGAGGAGGTGAGC
PFKM^a^	TGACCTCCAGAAAGCAGGTAAG	AACCAGGCCCACAATGTTC
GAPDH^a^	AAGGCTGGGGCTCATTTG	ACGAACATGGGGGCATC
CPT2^b^	AGCAGATGATGGTTGAGTGC	TCAAAGCCCTGGCCCATTG
SLC25^b^	GCATTGCAGGGATCTTCAACTG	ATATTTCCCAGGAGGTGCAGTC

LDHA, lactate dehydrogenase A; LDHB, lactate dehydrogenase B; MCT1, monocarboxylate transporter 1; MCT4, monocarboxylate transporter 4; PFKM, phosphofructokinase; GAPDH, glyceraldehyde 3‐phosphate dehydrogenase; CPT2, carnitine palmitoyltransferase 2; SLC 25, carnitine/acylcarnitine translocase, member 20.

a Genes involved in anaerobic energy metabolism.

b Genes involved in fatty acid oxidation.

### Statistics

All data in the text, figures, and tables are presented as mean ± standard deviation, unless otherwise stated. Prior to statistical testing, gene expression were log2‐transformed to maximize the likelihood of normal distribution.

Unpaired students t‐tests were used to test for differences between groups at pre and post, and differences in changes from pre to post, except for evaluating responses during the prolonged trials. Within‐group analyses were performed using paired t‐tests except for evaluating responses during the prolonged trials.

To evaluate changes in responses during the prolonged trials within groups (pre to post) a two‐way repeated measures analysis of variance (ANOVA) (time of intervention period and time during the prolonged trials as factors) with Sidek‐Holm post hoc test was performed. To evaluate differences in changes in the responses during the prolonged trials between the groups, a two‐way repeated measures ANOVA (changes from pre to post in each group and time point during the prolonged trial as factors) with Sidek‐Holm post hoc test were performed.

In addition, effect sizes for the key performance and physiological adaptations were calculated to compare the practical significance between the two groups. Effects size were calculated as Cohen's d and the criteria to interpret the magnitude were the following: 0–0.2 =  trivial, 0.2–0.6 =  small, 0.6–1.2 =  moderate, 1.2–2.0 =  large, and ˃2 =  very large (Hopkins et al. 2009).

Correlations analyses were done using the Pearson product‐moment method and correlations coefficients were interpreted according to Hopkins et al. (2009); *r* ˂ 0.1 trivial, 0.1–0.3 =  small, 0.3–0.5 =  moderate, 0.5–0.7 =  large, 0.7–0.9 =  very large, 0.9 =  nearly perfect, and 1.0 =  perfect.

Analyses were performed in GraphPad Prism 6 (GraphPad Software Inc., CA) and Excel 2013 (Microsoft Corporation, Redmon, WA). All analyses resulting in *P* ≤ 0.05 were considered statistically significant.

## Results

There were no significant differences between *E + S* and *E* at preintervention in any of the measured variables.

### Body mass, maximal strength, and leg_LM_

Body mass remained unchanged in *E+S* (pre: 62.4 ± 5.2 kg, post: 63.1 ± 5.6 kg), but was slightly reduced in *E* (pre: 65.6 ± 8.4 kg, post: 64.8 ± 8.0 kg *P* < 0.05). The change in body mass was different between groups (*P* < 0.05).

**Figure 1:**
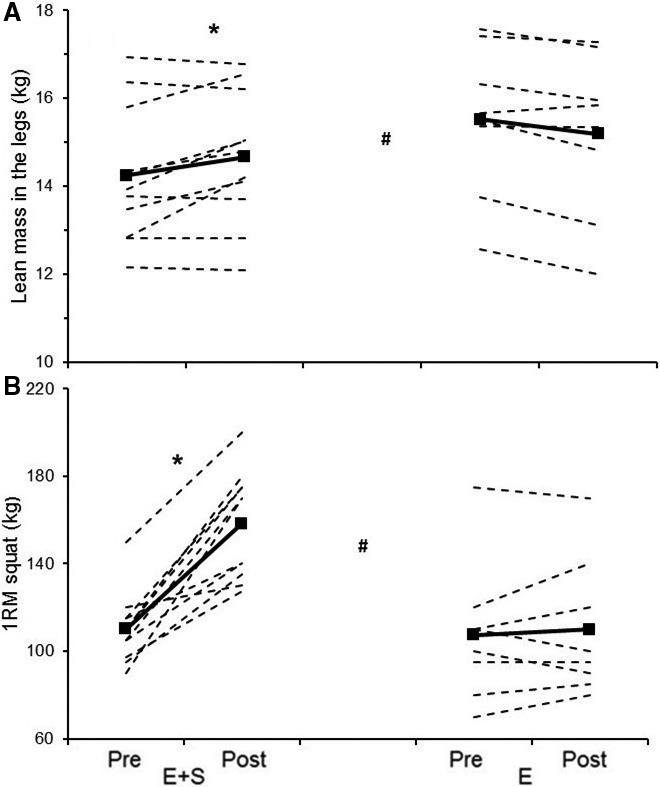
Individual values (dotted lines) and mean values (solid lines) before (Pre) and after (Post) the intervention period for athletes adding strength training to their normal endurance training (*E+S*,* n* = 11) and athletes performing normal endurance training only (*E*,* n* = 8). A: Lean mass in the legs. B: one repetition maximum (RM) in squat. * Different than pre (*P* ˂ 0.05), # the percent change from pre is different in *E + S* than in *E* (*P* ˂ 0.05).

*E + S* increased 1RM in half squat with 45 ± 22% (*P* < 0.01), while no change occurred in *E* (3 ± 10%, *P* = 0.52, Fig. 1). The change in 1RM was larger in *E + S* than in *E* (*P* < 0.01) and the ES analysis revealed a very large practical effect of *E + S* compared to *E* (ES = 2.4).

Leg_LM_ increased in *E + S* with 3.1 ± 4.0% (*P* < 0.05), while it decreased in *E* with 2.2 ± 2.1% (*P* < 0.05, Fig. 1). The change in leg_LM_ was larger in *E + S* than in *E* (*P* < 0.01) with a large practical effect of *E + S* compared to *E* (ES = 1.69).

Because of the reduced body mass in *E*, all *V*O_2_ measurements are presented as body mass adjusted values. Since power output measured using cycling ergometers does not correctly reflect the influence of body mass on outdoor cycling performance, especially during uphill cycling (Anton et al. 2007), power outputs measurements are reported as body mass adjusted values (W·kg^−1^). However, running at a treadmill is influenced by body mass to the same degree as outdoor running (McMiken and Daniels 1976); so, no body mass adjustments are done on the reported running distances.

### Muscle fiber type composition

The effect of the present intervention on fiber type composition has been previously reported (Vikmoen et al. 2016a). In brief, there was a reduction in the proportions of fibers positive for both IIA and IIX MyHC from 9 **±** 7% to 0% in *E+S* (*P* < 0.01) with a concomitant increase in type IIA fibers proportions from 39 **±** 13% to 51 **±** 10% (*P* < 0.01).

### Gene expression

**Figure 2:**
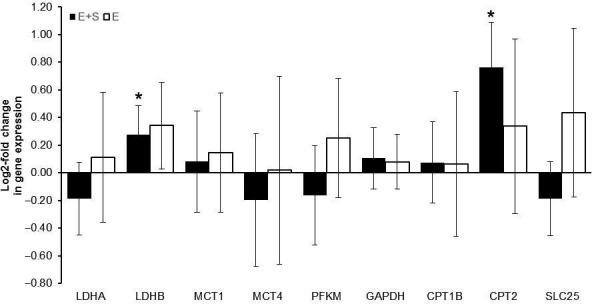
Log2‐fold change in mRNA expression for genes involved in fat transport and anaerobic metabolism during the intervention period for athletes adding strength training to their normal endurance training (*E + S*,* n* = 11) and athletes performing normal endurance training only (*E*,* n* = 8). * Different than pre (*P* ˂ 0.05). Values are mean ± 95% CI.

Of the nine genes investigated, only mRNA levels for CPT2 and LDHB increased 1.8 **±** 0.5 **‐**fold and 1.2 **±** 0.3–fold, respectively, in *E + S* (*P* < 0.05). The remainder of the genes did not change expression in response to the intervention (Fig. 2).

### *V*O_2max_ and *W*_max_/*V*_max_

The effect of the intervention used in this study on *V*O_2max_ and *W*_max_/*V*_max_ has been previously described (Vikmoen et al. 2016a,b). In brief, VO_2max_ in both cycling and running and *W*_max_/*V*_max_ were unchanged in both groups during the intervention period.

### Responses during the prolonged trials

**Figure 3:**
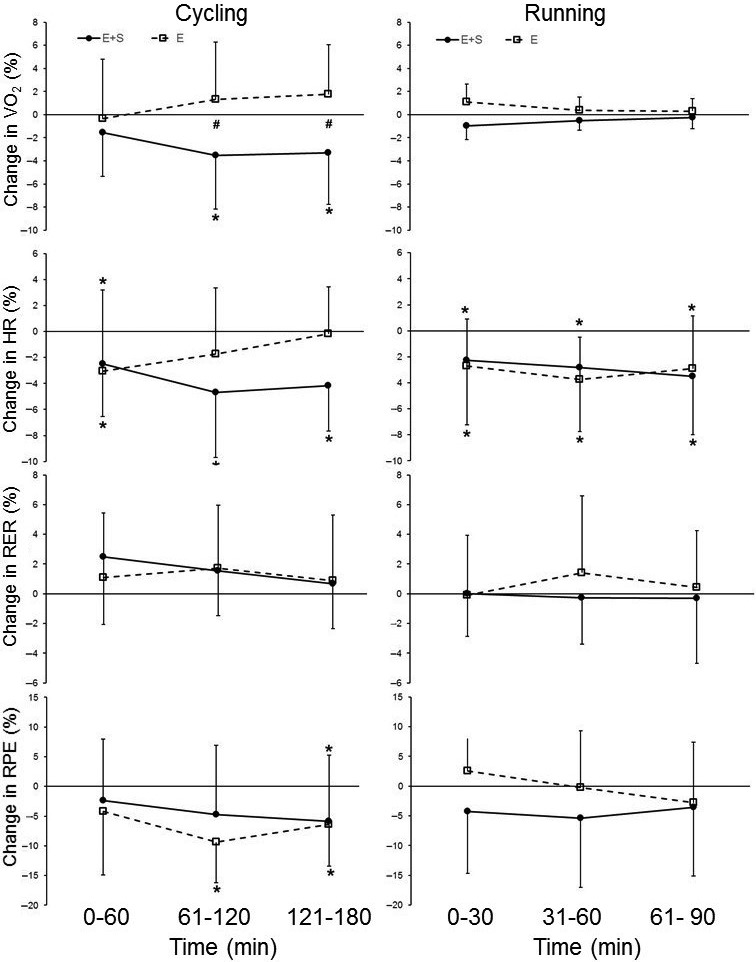
Percent change in responses during the prolonged trials in cycling (left panels) and running (right panels) for athletes adding strength training to their normal endurance training (*E + S*,* n* = 10) and athletes performing normal endurance training only (*E*,* n* = 8). Values are mean ± SD. * Different than pre (*P* ˂ 0.05), # the percent change from pre is different in *E + S* than in *E* (*P* ˂ 0.05).

The physiological responses during the prolonged trials are displayed in Table 2 and their percent changes are displayed in Figure 3. After the intervention, *E + S* reduced *V*O_2_ during the last two hours of the prolonged cycling trial (*P* ˂ 0.05) with no changes in *E*. The changes during the last two hours were different between the groups (*P* ˂ 0.05). In addition, the effect size analysis revealed a large practical effect of *E + S* compared to *E* during the last hour of the trial (ES = 1.2). There were no changes in VO_2_ for neither *E + S* nor *E* during the prolonged running.

**Table 2:** Responses during the prolonged trials in cycling and running for athletes adding strength training to their normal endurance training (*E + S*,* n* = 10) and athletes performing normal endurance training only (*E*,* n* = 8)

			*E+S*			*E*		
	Test section		First section	Middle section	Last section	First section	Middle section	Last section
VO_2_ (ml∙kg^−1^∙min^−1^)	Cycling	Pre	30.5 ± 2.9	31.3 ± 3.0	31.9 ± 2.9	30.1 ± 3.2	30.5 ± 3.4	31.0 ± 3.1
		Post	30.0 ± 2.5	30.2 ± 2.9^a^^,^ b	30.9 ± 3.2^a^^,^ b	29.9 ± 2.4	30.8 ± 2.9	31.5 ± 3.0
	Running	Pre	37.3 ± 1.8	37.7 ± 1.8	37.7 ± 1.8	37.0 ± 2.1	37.3 ± 2.0	37.3 ± 1.8
		Post	37.0 ± 2.2	37.5 ± 2.0	37.6 ± 1.9	37.4 ± 2.0	37.4 ± 1.5	37.4 ± 1.4
HR (beats∙min^−1^)	Cycling	Pre	134 ± 12	138 ± 14	143 ± 14	129 ± 11	130 ± 9	135 ± 7
		Post	131 ± 12^a^	131 ± 14^a^	137 ± 13^a^	125 ± 9^a^	128 ± 10	135 ± 9
	Running	Pre	158 ± 12	163 ± 13	165 ± 13	152 ± 11	157 ± 11	158 ± 11
		Post	154 ± 11^a^	158 ± 10^a^	159 ± 11^a^	148 ± 13^a^	151 ± 11^a^	153 ± 11^a^
RER	Cycling	Pre	0.85 ± 0.03	0.84 ± 0.03	0.82 ± 0.03	0.87 ± 0.03	0.84 ± 0.03	0.81 ± 0.04
		Post	0.87 ± 0.04	0.85 ± 0.03	0.82 ± 0.03	0.88 ± 0.03	0.85 ± 0.03	0.82 ± 0.03
	Running	Pre	0.90 ± 0.02	0.89 ± 0.02	0.88 ± 0.02	0.90 ± 0.02	0.87 ± 0.03	0.86 ± 0.03
		Post	0.91 ± 0.03	0.88 ± 0.03	0.86 ± 0.03	0.90 ± 0.02	0.88 ± 0.03	0.86 ± 0.03
RPE (Borg scale)	Cycling	Pre	11 ± 1	12 ± 1	13 ± 1	11 ± 2	12 ± 2	13 ± 2
		Post	11 ± 1	12 ± 1	12 ± 1^a^	10 ± 2	11 ± 1^a^	12 ± 1^a^
	Running	Pre	12 ± 1	13 ± 1	13 ± 1	11 ± 2	12 ± 1	13 ± 1
		Post	11 ± 1	12 ± 1	13 ± 1	11 ± 1	12 ± 1	13 ± 1
Cadence (rev∙min^−1^)	Cycling	Pre	84 ± 8	83 ± 10	83 ± 10	83 ± 10	81 ± 12	80 ± 13
		Post	85 ± 9	83 ± 8	83 ± 9	81 ± 11	81 ± 12	80 ± 14
	Running	Pre	–	–	–	–	–	–
		Post	–	–	–	–	–	–

Values are mean ± SD.

a Different than pre (*P* ˂ 0.05)

b The change from pre to post is different in *E+S* than in *E* (*P* ˂ 0.05).

*E + S* had a reduced HR throughout the prolonged cycling after the intervention (*P* ˂ 0.05), while *E* had a reduced HR during the first hour only (*P* ˂ 0.05). There was a moderate practical effect of *E + S* compared to *E* during the last hour of the trial (ES = 1.12). The correlation between changes in *V*O_2_ and HR during the last hour of the prolonged cycling was large (*r* = 0.59). Both *E+S* and *E* had a reduced HR during the entire prolonged running trial after the intervention period (*P* ˂ 0.05). There was no difference in changes between the groups.

Compared to the pretrial, RPE was lower during the last hour of prolonged cycling for *E + S* and lower during the last two hours for *E* (*P* ˂ 0.05). However, there were no differences in changes between the groups. RPE did not change during the prolonged running. There were no changes in RER in neither of the groups during the prolonged trial in both cycling and running. In cycling, cadence did not change in either group during the intervention.

### 5‐min all‐out tests

**Figure 4:**
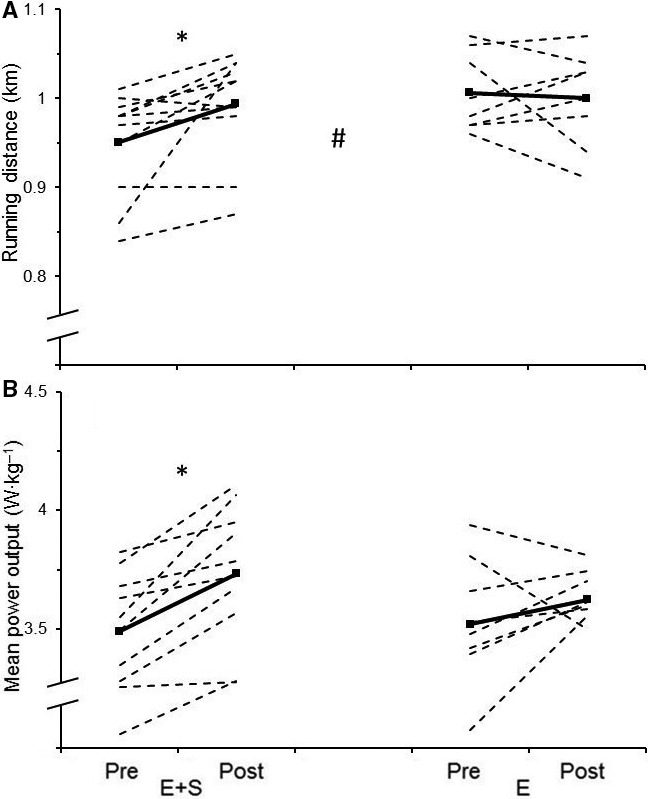
Individual values (dotted lines) and mean values (solid lines) before (Pre) and after (Post) the intervention period for athletes adding strength training to their normal endurance training (*E + S*,* n* = 10) and athletes performing normal endurance training only (*E*,* n* = 8). A: Running distance during the 5‐min all‐out running test. B: Mean power output during the 5‐min all‐out cycling test. * Different than pre (*P* ˂ 0.05), # the percent change from pre is different in *E + S* than in *E* (*P* = 0.05).

**Figure 5:**
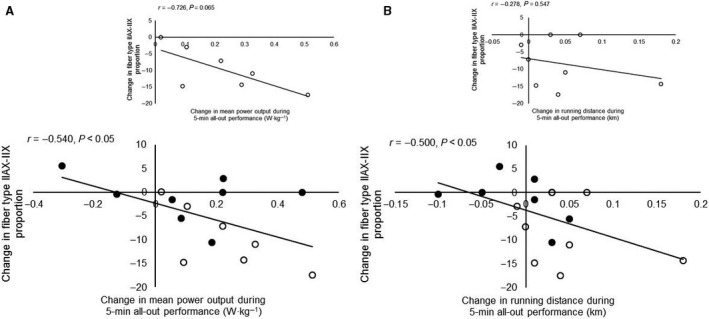
A: Correlation between changes in type IIAX‐IIX proportions and changes in mean power output during the 5‐min all‐out cycling test. The inserted panel shows the correlation when only the athletes adding strength training to their normal endurance training are included. B: Correlation between changes in type IIAX‐IIX proportions and changes in running distance during the 5‐min all‐out running test. The inserted panel shows the correlation when only the athletes adding strength training to their normal endurance training are included.

After the intervention, the mean power output during the 5‐min all‐out cycling test increased by 7.0 ± 4.5% (*P* < 0.05) in *E+S* with no change in *E* (3.3 ± 7.1%, *P* = 0.27 Fig. 4). The difference between the groups was not statistically significant, but the practical effect of *E + S* compared to *E* was moderate (ES = 0.62). *E + S* increased running distance in the 5‐min all‐out running test by 4.7 ± 6.0% (*P* < 0.05) with no change in *E* (−0.6 ± 5.0%, Fig. 4). The increase in running distance was larger in *E + S* than in *E* (*P* = 0.05), and the practical effect of *E + S* compared to *E* was moderate (ES = 0.95). Correlation analyses revealed a large correlation between change in all‐out cycling performance and W_max_ (*r* = 0.54, *P* ˂ 0.05) and between all‐out running performance and *V*_max_ (*r* = 0.53, *P* ˂ 0.05). There was a large correlation between change in all‐out performance and the training induced change in IIAX‐IIX fibers in cycling (*r* = −0.54, *P* ˂ 0.05, Fig. 5) and in running (*r* = −0.50, *P* ˂ 0.05, Fig. 5) when data from both groups were included. When only *E + S* athletes were included, the correlation got very large in cycling (*r* = −0.73, *P* = 0.065, Fig. 5) but disappeared in running (*r* = −0. 28, *P* = 0.547, Fig. 5). The correlation between the percent change in running distance and mean power output in cycling was moderate but not statistically significant (*r* = 0.40, *P* = 0.10).

## Discussion

The main finding of this study is that addition of heavy strength training to the regular endurance training of female duathletes improved both running and cycling performance measured as 5‐min all‐out performance tested immediately after prolonged submaximal work. In addition, VO_2_ and HR were reduced during the last two hours of a 3‐h prolonged cycling trial after the addition of heavy strength training, whereas no effects of added strength training were observed on physiological responses during prolonged submaximal running.

### Strength, leg_LM_, and muscle fiber type composition

The observed increase in 1RM in half squat and leg_LM_ is in accordance to previously observed improvements in endurance athletes adding 8–12 weeks of heavy strength training (e.g., Bishop et al. 1999; Storen et al. 2008; Ronnestad et al. 2010a; Aagaard et al. 2011; Ronnestad et al. 2015). The results lend further support to the notion that a substantial increase in strength can be achieved with little or no change in body mass (Storen et al. 2008; Ronnestad et al. 2010a; Sunde et al. 2010; Ronnestad et al. 2015). Increased body mass is usually undesirable for performance in cycling and running and therefore a concern among endurance athletes considering adding strength training. The increase in leg_LM_ reported in this study indicates that at least some of the improved strength was due to muscle hypertrophy. In addition, we observed a fiber type shift from type IIAX‐IIX toward type IIA fibers (Vikmoen et al. 2016a), a common adaptation to strength training among both untrained and endurance trained individuals (Staron et al. 1994; Aagaard et al. 2011). The increased leg_LM_ and fiber type shift shows that the strength training program was effective in inducing adaptations at the muscular level.

### Physiological responses during the prolonged trials

As previously observed in well‐trained male cyclists (Ronnestad et al. 2011), *E+S* reduced *V*O_2_ during the last two hours of the prolonged cycling after the strength training intervention. Therefore, although no change in cycling economy was observed during the first hour, cycling economy was clearly improved when the athletes started to get fatigued. This is highly important in cycling where many races include prolonged submaximal intensities for several hours. Improved cycling economy have also been reported in untrained individuals (Loveless et al. 2005) and trained male cyclists (Sunde et al. 2010) after strength training interventions when measured in a nonfatigued state. However, this seems not to be the case in highly trained to elite cyclists (Ronnestad et al. 2010a; Aagaard et al. 2011; Ronnestad et al. 2015). The results from this study and the study by Ronnestad et al. (2011) indicate that after a strength training intervention, cycling economy should also be tested when the athletes are somewhat fatigued.

HR was reduced throughout the prolonged cycling trial after the intervention period in *E + S* and as for *V*O_2_ the effect was more pronounced during the last 2 h. Consequently, the reduced HR was probably because of the reduced *V*O_2_ and hence reduced energy cost. In fact, the reduced HR mirrored the changes in *V*O_2_ and a large correlation between change in *V*O_2_ and change in HR during the last hour was observed (*r* = 0.59).

The mechanisms behind improved cycling economy during the last 2 h of the trial are somewhat unclear. One explanation might be delayed recruitment of type II muscle fibers brought on by increased muscle strength and muscle mass (Ronnestad et al. 2011). When the maximal muscle strength increases and the absolute power output and cadence remains the same, the level of force developed in each pedal thrust is reduced relatively to the maximal force. Given the size principle of motor unit recruitment, this implies that the more economical type I muscle fibers can account for a larger proportion of the same absolute power output (Hickson et al. 1988; Ronnestad and Mujika 2014). This may also explain the lack of changes in cycling economy during the first hour where the relative low power output should mainly recruit type I muscle fibers, thereby leaving little potential for improvements. In fact, it has previously been reported that after exercise for 60 min at an intensity requiring 43% of *V*O_2max_, glycogen breakdown mainly occurred in the type I muscle fibers (Vollestad and Blom 1985), indicating limited recruitment of type II fibers. However, as the duration of the work increases and muscle fibers starts to get fatigued, additional motor units needs to be recruited to sustain the power output (Gollnick and Armstrong 1973; Vollestad and Blom 1985). The suggested mechanisms is therefore that the strength training allowed *E + S* to use the more economical type I muscle fibers for a longer duration of the trial after the intervention, leading to improved cycling economy during the last part. Supporting this, 5 weeks of strength training has been shown to reduce EMG activity in *m. vastus lateralis* during the last hour of a 2‐hour prolonged cycling trial in well‐trained triathletes (Hausswirth et al. 2010).

The fiber type transition from type IIAX‐IIX to type IIA in *E+S* might also contribute to the improved cycling economy since it has been suggested that type IIA fibers are more economical than the type IIX fibers (Westerblad et al. 2010). However, there was no correlation between the changes in the proportions of type IIAX‐IIX and changes in economy during the last hour of the prolonged cycling. This may be because the relatively low power output did not recruit any type IIX fibers during the trial even before the intervention.

Other possible explanations for improved cycling economy during the last 2 hours of the prolonged cycling trial could have been changes in substrate utilization toward larger carbohydrate utilization (Mogensen et al. 2006) or reduction in cadence (Foss and Hallen 2004). However, there were no changes in RER or cadence during the prolonged cycling, making these explanations unlikely. In fact, based on the increased mRNA levels of CPT2, a protein involved in fatty acid oxidation in the mitochondria, an increased utilization of fat as an energy substrate might have been expected. However, in Vikmoen et al. (2016a), we did not find changes in the content of the beta‐oxidation enzyme hydroxyacyl‐CoA dehydrogenase (HADH) in the very same biopsy material, supporting the notion that rates of fatty acid oxidation did not change.

In contrast to cycling, no changes occurred in *V*O_2_ during the prolonged running. This is surprising since the proposed mechanisms for the reduced *V*O_2_ during cycling in theory also could reduce *V*O_2_ during the prolonged running. However, some methodological differences might explain the different finding between cycling and running. The prolonged running was only half as long as the prolonged cycling and was performed at a higher relative workload (60% vs. 44% of *V*_max_ and *W*_max_, respectively). Because the reduced *V*O_2_ during the cycling trial was seen during the last 2 h, it may be speculated that the prolonged running were too short. However, running races do seldom last as long as cycling races, and the shorter duration was therefore chosen for the prolonged running. To compensate for the shorter duration, the prolonged running was performed at a higher relative intensity than the prolonged cycling. This may have led to a quite high recruitment of type II motor units from the start, and the potential for reduced *V*O_2_ during the last part of the trial may therefore have been limited. In fact, in a glycogen breakdown study, it was estimated that a large proportion of type IIA fibers were recruited already from the start at a power output corresponding to 61% of *V*O_2max_ (Vollestad and Blom 1985).

No changes in running economy after addition of strength training is in conflict with results from previous studies where improved running economy ranging from 3 to 8% have been reported (e.g., Paavolainen et al. 1999; Storen et al. 2008; Sedano et al. 2013). Some methodological differences might explain this discrepancy. Running economy was tested with an inclination of 5.3% in our study, and in combination with the relative low workload, the velocity during the prolonged running was quite low compared to previous studies. In fact, the improvements in running economy after strength training have been reported to be dependent on running velocity (Saunders et al. 2006). The lack of effect on running economy may also be because the strength training program used did not induce any changes in patellar tendon stiffness (Vikmoen et al. 2016b). Changes in muscle‐tendon stiffness is a frequently proposed mechanism behind improved running economy after strength training (Saunders et al. 2006; Storen et al. 2008).

### Performance during the 5‐min all‐out tests

The improved cycling performance observed in the 5‐min all‐out test is in accordance with a similar study in male cyclists, who found increased 5‐min all‐out performance following prolonged cycling after adding strength training to their normal endurance training (Ronnestad et al. 2011). A novel finding in this study is that 5‐min all‐out running performance after a prolonged submaximal trial also seems to be affected to the same degree as in cycling. Improved running and cycling performance after strength training is in accordance with previous studies in cycling (Koninckx et al. 2010; Ronnestad et al. 2010a,b; Sunde et al. 2010, Aagaard et al. 2011; Ronnestad et al. 2015; Vikmoen et al. 2016a) and running (Paavolainen et al. 1999; Storen et al. 2008; Sedano et al. 2013; Damasceno et al. 2015) when performance is measured in a more traditional way. However, other studies contradict these findings both in cycling (Bishop et al. 1999; Bastiaans et al. 2001; Levin et al. 2009) and running (Ferrauti et al. 2010; Roschel et al. 2015). Some methodological differences may explain these equivocal findings. To positively affect cycling performance, it seems that the strength training regime needs to involve heavy training load (4‐10RM), rather large volumes of training and last for 8 weeks or longer (Koninckx et al. 2010; Ronnestad et al. 2010a, Aagaard et al. 2011; Ronnestad et al. 2015). On the other hand, both explosive, plyometric and heavy strength training seems effective in improving running performance (Paavolainen et al. 1999; Storen et al. 2008; Sedano et al. 2013; Damasceno et al. 2015).

Together, these observations indicate that the mechanisms behind changes in running and cycling performance after strength training may be somewhat different. However, improvements in both cycling and running performance may be related to typical adaptations to prolonged periods of heavy strength training such as increased muscle mass and fiber type transitions from type IIX to type IIA; improvements in running performance may also rely on adaptations such as changes in leg stiffness, rate of force development, and other neuromuscular characteristics. Therefore, mechanisms behind the improved performance in cycling and running in this study might be different. This is supported by the fact that the correlation between changes in running and cycling performance (*r* = 0.40) were not statistically significant.

Since the performance tests were performed right after the prolonged trials, changes in the physiological responses to the submaximal exercise was expected to affect performance. We suggest that the reduced *V*O_2_ and HR observed during the last 2 hours of the cycling trial, indicating reduced physiological strain and less fatigue, made the athletes in *E + S* capable of producing higher mean power output during the final 5‐min all‐out test. Furthermore, reduced *V*O_2_ in *E + S* means that the total energy consumption during the prolonged cycling trial was lower after the intervention and with no change in substrate utilization the total carbohydrate utilization was reduced. Therefore, some of the improved cycling performance in *E + S* may be due to a better conservation of glycogen stores during the prolonged trial. The importance of less physiological strain during the submaximal exercise is indirectly supported by the fact that 5‐min all‐out performance, tested in the rested state, was unchanged after 16 weeks of strength training in elite cyclists (Aagaard et al. 2011).

Based on the present data, the positive effect of strength training on performance in the 5‐min all‐out running test cannot be explained by changes in physiological responses during the submaximal running. Therefore, the improved running performance after strength training has to be through other mechanisms. During a 5‐min all‐out test a substantial part of the energy is derived from anaerobic metabolism (Gastin 2001). Therefore, increased anaerobic capacity might be a mechanism behind the improved performance in both cycling and running. In fact, endurance performance has been reported to correlate well with measurements of anaerobic performance (e.g., Bulbulian et al. 1986; Houmard et al. 1991). Increased anaerobic capacity can be achieved through increases in muscle mass (Bangsbo et al. 1993) and/or through increasing amount of anaerobic enzymes. Even though small to none changes were found in mRNA expression of genes coding for important proteins in anaerobic metabolic pathways in *E + S*, the increased muscle mass should mean that anaerobic capacity was improved. Anaerobic capacity should also affect performance in *V*_max_/*W*_max_. Even though there were no significant changes in these variables, the correlation between changes in *V*_max_ and running performance and *W*_max_ and cycling performance further support that improved anaerobic capacity might play a role for the improved performance in *E + S*. In addition, there was a very large correlation between leg_LM_ and absolute average power output during the 5‐min all‐out test before the intervention (*r* = 0.71, data not shown) indicating that muscle mass is important in these kinds of tests.

There were large correlations between the reduction in muscle fiber type IIAX‐IIX proportions and changes in 5‐min all‐out performance in both cycling and running. The type IIA fibers is less fatigable than the type IIX fibers (Westerblad et al. 2010), and a fiber type transition could therefore improve performance. However, a correlation between two variables does not necessarily mean a cause and effect relationship (Greenfield et al. 1998). Perhaps, the athletes with a large reduction in fiber type IIAX‐IIX proportions had a large response to the strength training and that other adaptations to the strength training actually were responsible for the improved performance. Indeed, there was a large negative correlation (*r* = −0.65, data not shown) between the change in leg_LM_ and change in the proportion of type IIAX‐IIX fibers. Notably, when only *E+S* was included, the correlation between 5‐min all‐out performance and IIAX‐IIX fiber transitions got very large in cycling and disappeared in running. This indicates that the possible performance‐enhancing effects from fiber type shift from type IIAX‐IIX toward type IIA was more important in cycling than in running.

The improved performance cannot be explained by changes in *V*O_2max_ since *V*O_2max_ did not change in neither cycling nor running. The lack of effect of strength training on *V*O_2max_ is not surprising and is in accordance with the current literature (e.g., Storen et al. 2008; Aagaard et al. 2011; Ronnestad et al. 2015). Importantly, we expected no change in *V*O_2max_ in the study, as athletes were instructed to continue their normal endurance training, having a good base of training from their winter training consisting of running, cycling, and cross‐country skiing.

This is the first controlled study to demonstrate that adding heavy strength training to endurance training leads to improvements in both cycling and running performance in the same athletes. Performance was tested as 5‐min all‐out performance, measured immediately after prolonged periods of submaximal work. The improved cycling performance was probably related to reduced physiological strain during the submaximal trial. This is also the first study reporting improved running performance following a prolonged submaximal effort. However, there were no changes in the physiological responses to prolonged running. Therefore, improved running performance was more likely related to other mechanisms like changes in anaerobic capacity and neuromuscular changes. Changes in anaerobic capacity probably also contributed to improved cycling performance. A fiber type shift from type IIAX‐IIX toward type IIA in the main propulsive muscles also seemed to contribute to the improved performance, especially in cycling. Based on the results of this study, both runners and cyclists should include heavy strength training in their training programs for maximal gains in performance. This seems to be particularly important for performance during late phases of long‐lasting competitions.

## Conflict of Interests

None declared.
